# Microbial Diversity of Spontaneously Fermented Camel Milk

**DOI:** 10.3390/foods15111969

**Published:** 2026-06-02

**Authors:** Mudhi A. Abaalkhail, Sahar H. S. Mohamed, Mohammed S. Aljurbua, Raghad A. Alkhuraisi, Mohammed Aladhadh

**Affiliations:** Department of Food Science and Human Nutrition, College of Agriculture and Food, Qassim University, Buraydah 51452, Saudi Arabia

**Keywords:** camel milk, fermentation, lactic acid bacteria, microbial diversity, pathogens, culture-dependent and culture-independent methods

## Abstract

Camel milk is widely consumed in the world’s arid and semi-arid regions because of its favorable nutritional profile and associated human health benefits. The indigenous microbiota of raw camel milk is diverse and composed of different bacterial and fungal groups. This community drives spontaneous milk fermentation, resulting in a variety of traditional products, including Gariss, Shubat, Chal, Dhanaan, Lfrik, and Suusac (or Suusa), depending on geographic region and cultural practice. This fermented milk has improved sensory, nutritional, and health profiles, as well as an extended shelf life, compared to raw milk. Fermentation alters the microbial community structure, with lactic acid bacteria (LAB) consistently becoming dominant, while yeasts and molds are also detected in some products. These patterns have been identified using both culture-dependent and culture-independent approaches, including 16S rRNA gene sequencing and whole-genome shotgun metagenomics. However, the milk’s microbial composition is highly variable and is influenced by the original composition, geographical location, fermentation and hygiene practices. The detection of opportunistic pathogens such as *E. coli*, *Salmonella* and *Listeria* in some traditional products raises important food safety concerns. This review presents current knowledge on fermented camel milk microbiology using a cross-regional approach, identifying key gaps in microbial safety and process standardization to support wider acceptance and potential commercialization.

## 1. Introduction

Camel milk is known as the “desert white gold” and is widely consumed in the arid and semi-arid regions, especially among pastoral communities, because of its perceived unique nutritional and medicinal properties [1,2]. Camel milk is an essential component of the diet of nomads and is used in traditional medicine for the treatment of various diseases. It is believed to be effective in the treatment of tuberculosis, diabetes, liver disease, respiratory disorders, diarrhea, gallstones, nervous disorders, general fatigue, and gastric ulcers [2,3]. While raw camel milk may have different bacterial species (including pathogens) [4], it also contains potential probiotic bacterial strains [5], which can improve gut health, lower cholesterol, and improve milk nutrients’ availability. As a result of its physicochemical properties, safety profile and traditional association with disease treatment and health improvements, there is an increasing scientific and industrial interest in the study of camel milk to validate its nutritional and health improvement potentials.

Camel milk can be fermented spontaneously by its indigenous microbiota, producing different traditional milk products such as Lfrik [6], Gariss [7], Chal [8], and Dhanaan [9]. This fermentation is carried out by bacterial and fungal groups that produce organic acids, alcohol, and volatile compounds, enhancing the product’s texture, taste and safety [10]. Fermentation also causes a shift in the microbial community composition [11] and activities, which leads to improved product shelf life through acidification [12]. Acidification allows the milk to be easily stored at ambient temperatures, as low-temperature storage facilities like refrigeration are often not available for use by pastoralists. Camel milk also contains bioactive peptides, biogenic amines, vitamins, antioxidants and hypocholesterolemic compounds with reported antidiabetic, angiotensin-converting enzyme (ACE)-inhibitory, antidiarrhoeal, and anticancer activities [13,14,15]. Compared with bovine milk, Camel milk was found to contain lower saturated and higher unsaturated fatty acids, with higher ACE inhibitory potential compared to cow milk [16].

The microbial community in fermented camel milk is more diverse than that in the raw milk samples [17]. It is dominated by lactic acid bacteria (LAB), but may also contain poor hygiene indicator and psychrotrophic spoilage microorganisms [11,18]. The major groups of LAB involved in the fermentation include *Lactobacillus*, *Lactococcus*, *Leuconostoc*, and *Enterococcus* species [19]. An extensive list of the LAB and other microbial groups involved in the fermentation process is presented in Table 1. The acidification of the milk by LAB not only improves its shelf life but also inhibits pathogens [20]. However, it is important to note that the fermented milk’s microbial composition varies with hygiene, processing conditions, season, and geography [21]. Therefore, studying the microbial prevalence and dynamics in fermented camel milk is critical for ensuring the milk’s safety [22] and quality. Additionally, this knowledge aids the identification of probiotic strains and the optimization of the traditional fermentation process for product improvement [23,24].

Unfortunately, studies on camel milk are often fragmented, or product- or region-specific, with limited focus on pathogenic groups, microbial succession and associated safety issues. Therefore, this review synthesizes the current knowledge on fermented camel milk products’ diversity and the fermentation process across different geographical locations with a focus on their microbial composition and diversity deduced from culture-dependent and -independent studies. This should allow comparative analysis of microbial composition across fermented milk products. Furthermore, gaps in scientific knowledge can be identified and future research directions outlined to optimize the indigenous microbiota activities, for improved safety, nutritional profile and potential commercial prospects.

## 2. Fermentation as a Traditional Preservation Method of Camel Milk

Camel milk is commonly consumed raw or as a naturally fermented product by pastoralists, with milk fermentation [40] supporting milk preservation in desert conditions, where the cold chain/storage was often not available [41]. This process involves the conversion of lactose into lactic acid, facilitated by the natural microbiota present in milk [41]. The lactic acid fermentation process, which is beneficial to the milk’s shelf life and nutritional properties, is also a low-cost process that does not require significant operator expertise [36]. Traditionally, fermented camel milk is allowed to ferment naturally without any prior heat treatment and sometimes without supplementation with starter cultures [36]. The shelf life of fermented camel milk, although longer than that of unfermented milk, varies based on factors such as the initial microbial load, storage conditions, and processing methods [42]. Pasteurizing camel milk before fermentation is now known to improve its microbial content in addition to extending its shelf life [43].

### 2.1. Different Types of Fermented Camel Milk

In several countries across the world, fermented camel milk is produced by different communities with differences in the processing conditions, its microbiological, physicochemical, and chemical properties, and its volatile organic compound profiles. An example of such milks is Chal, which is typically made by adding a previously fermented acidic milk as inoculum in an earthenware jug for 1 or 2 days, depending on the season of production in Iran [8]. This drink has a low-calorie content and is rich in calcium, iron, zinc and magnesium [5]. Suusac (or suusa) is another example, and this is a traditional fermented camel milk produced in Kenya and Somalia, by incubating camel milk in smoked wooden buckets for up to 3 days [44]. The camels are milked directly into a gourd that has been cleaned, smoothed and treated with smoke. The smoking of the gourd is performed using smoldering twigs of the acacia tree (*Acacia seyal*). The smoking is believed to improve the quality of Suusac (suusa), giving it its characteristic flavor and aroma, while improving its color. After fermentation, the top fatty layer is removed and the product is ready for consumption for up to a week at room temperature (26–29 °C) [45]. Gariss is a fermented camel milk popular among the nomads of Sudan, which is prepared by naturally fermenting the camel milk in large skin bags that contain a large quantity of a previously soured product. Fermentation is carried out at ambient temperature without any heat treatment [26]. In addition to the activities of the indigenous microbiota, fermentation of Gariss is reported to be initiated by the addition of black cumin seed and onion bulbs [34]. Moreover, the continuous agitation during the nomadic transport process is thought to significantly enhance the fermentation efficiency [46].

Dhanaan is the name for fermented camel milk in Eastern Ethiopia, made by placing unpasteurized camel milk in a smoking container, wrapping it in cloth, and leaving it at ambient temperature (25–35 °C) for an extended period. The milk has a white, opaque color, a sour flavor, and a thin consistency [47]. Smoking the milk containers with specific wood species is common, and pastoralists claim that smoking extends the product’s shelf life and improves its taste and aroma. Pastoralists further extend Dhanaan’s shelf life by frequently adding fresh milk [48]. Lfrik is another traditional fermented camel milk produced in Morocco, typically made by naturally fermenting unpasteurized camel milk in goat skin bags known as “Tassoufra” for approximately 12 h at ambient temperature [6]. Shubat is a traditional fermented camel milk produced in Kazakhstan, typically made by adding a small quantity of previously soured milk as an inoculum to fresh raw camel milk. Fermentation is carried out for 1 to 2 days in specialized containers made of skin or wood at ambient temperature [19,38]. These wooden vessels function as efficient natural inoculation systems, with porous surfaces that harbor biofilms releasing large populations of lactic acid bacteria into the milk upon contact [25].

### 2.2. The Fermentation of Camel Milk

In general, the study of the fermentation of dairy products involves monitoring the diversity and activities of microorganisms involved in the fermentation process and changes in the milk’s physicochemical parameters, such as changes in pH and milk proteins (coagulation) [43]. The acidification process in camel milk is characteristically slower than in cow milk, with research indicating that acidity levels in cow milk can reach twice the values observed in camel milk under identical fermentation conditions [47]. This delayed fermentation rate is primarily attributed to the high concentrations of natural antimicrobial inhibitors present in camel milk, including lysozyme, lactoferrin, lactoperoxidase, and immunoglobulins, which are present at significantly higher levels than in bovine milk [43]. The fermentation of camel milk induces a significant microbial shift, transitioning from a diverse raw state to a specialized environment dominated by lactic acid bacteria (LAB). This is supported by increases in LAB populations. For example, the initial viable counts of starter cultures (4.39–4.7 log10 CFU/mL) increased substantially, reaching peak concentrations of 6.71 to 8.2 \log10 CFU/mL after 6 h of fermentation [48]. This substantial increase confirms the rapid proliferation and predominance of LAB as the primary microbiota in the final fermented product [25]. Despite the high biodiversity of camel milk microbiota, starter cultures used in the camel industry are mainly taken from bovine milk [41]. However, for any commercial development of camels’ fermented milk, there will be a need to have standardized/optimized fermentation conditions to produce milk with the desired organoleptic properties. The public health benefit of such products due to their potential probiotic effect would have to be evaluated as well as any potential public health risks which may be associated with potential pathogenic groups.

## 3. Microbial Diversity in Raw and Fermented Camel Milk Products

Culture-based assays have identified different bacterial groups such as *Enterobacter*, *Lactococcus*, *Enterococcus*, *Lactobacillus*, *Staphylococcus*, and *Escherichia*/*Shigella* in raw camel milk [18]. Similarly, the next-generation sequencing (NGS) of raw camel milk from China showed that it was dominated by *Epilithonimonas*, with groups belonging to *Acinetobacter* sp. and *Klebsiella* were also detected [49]. Other NGS-based assays have identified *Lactobacillus*, *Sphingomonas*, *Paenibacillus*, *Streptomyces* and *Shigella* as the dominant bacterial groups in raw camel milk [21,50]. Fungal groups such as *Penicillium*, *Cladosporium*, *Candida*, *Aspergillus*, *Alternaria* and *Fusarium* have been detected in raw camel milk [21]. The LAB found in fermented camel milk is dominated by mesophilic species. The pathogenic bacterial species in camel milk include coliforms, *Staphylococcus*, *Enterobacter*, *Salmonella*, *Listeria*, and *E. coli*, whose prevalence varies depending on the environmental conditions and the hygienic conditions of the farm [43].

During traditional preparation of Dhanaan, the milk is kept in sealed containers, suggesting that the microbial flora driving the acidification and fermentation process likely consists of thermophilic anaerobic microorganisms [51]. The study recorded ethanol levels in Gariss of up to 1.40% as a result of yeast activity, which indicated that Gariss undergoes yeast–lactic fermentation [52], although higher ethanol levels have been recorded in other studies (Table 1). Multivariate statistical analyses indicated that the bacterial microbiota composition of naturally fermented milk is significantly shaped by both its geographic origin and sample type [53]. Fermentation, whether spontaneous or controlled, selects for specific groups of lactic acid bacteria (LAB), yeasts, and molds, leading to microbial succession [27]. However, existing studies on fermented camel milk remain fragmented and employ different methodological approaches. This heterogeneity limits direct comparison across studies. To address this, the microbial profiles of selected traditional fermented camel milk products have been compiled into a unified table (Table 1), summarizing dominant LAB, yeasts, potential pathogens, and selected physicochemical parameters reported across different studies.

The microbiological analysis of Lfrik revealed the presence of high numbers of coliforms and enterococci and the absence of *Salmonella* and *Staphylococcus aureus* in the samples analyzed [47]. In contrast, the microbial community of Dhanaan samples was found to be a complex ecosystem dominated by *Proteobacteria*, particularly members of the *Enterobacteriaceae* family [9]. Similarly, the study concluded that ‘Chal’ contains a wide variety of yeast species that become predominant after 48 h of fermentation [54]. The high prevalence of *Enterobacteriaceae* indicates poor hygienic conditions and raises serious health concerns. The presence of *Acinetobacter* further suggests environmental contamination [9]. Overall, the presence of pathogenic and spoilage-associated bacteria alongside beneficial LAB indicates that traditional Dhanaan production practices may be microbiologically unsafe [9]. Lactating camels with mastitis also contribute to foodborne pathogens and, therefore, they can also serve as a source of pathogens [55]. The absence of pathogenic bacteria (*Salmonella* and *S. aureus*) in some camel milk may be due to their inhibition by LAB, through bactericidal bacteriocins or due to the acidity produced during the fermentation process [47]. Additionally, the absence of coliform bacteria in Gariss is attributed to its high acidity, with pH values ranging from 3.41 to 3.82 in some studies [46]. The poor microbiological quality of Suusac (or suusa) and its associated public health risks are driven by various factors along the informal production and market chain, including unhygienic handling, environmental cross-contamination, and inadequate production and storage practices [32,55]. The microbial load of Suusac (or suusa) increased along the value chain from production to market, indicating continuous contamination. This rise is mainly associated with poor handling practices, such as storage in unhygienic containers, mixing milk from different times or suppliers, raw milk consumption, and roadside selling, rather than fermentation alone [28]. Similarly, the results ultimately indicated that traditionally made Dhanaan is of substandard microbiological quality compared to laboratory-prepared versions. This poor quality is largely attributed to unhygienic practices during milking and handling, such as the use of untreated water and poor personal hygiene among producers [29]. Furthermore, the presence of these yeasts in a product made from raw camel milk (‘Chal’) suggests potential contamination from environmental sources such as air, water, or handling during preparation and transport. Therefore, improving hygienic standards during production is recommended to decrease the risk of yeast contamination [54]. The metagenomic analysis also detected minor genera, such as *Rahnella* and *Pseudomonas*; however, their functional roles remain unclear, and their growth is likely suppressed by the rapid acidification and competitive inhibition provided by the dominant lactic acid bacteria and yeasts during Shubat fermentation [12]. The detection of species such as Lactobacillus (*L. kefiranofaciens*) underscores the diverse microbial ecology of Shubat, which is influenced by regional variations in raw milk composition and specific fermentation practices across different areas [12]. According to [30], the Shubat microbiome is significantly distinctive compared to other traditional fermented milk products, characterized by the dominance of *Lentilactobacillus kefiri* and *Lactobacillus kefiranofaciens*, along with the first-time identification of *Bifidobacterium mongoliense* in this product [30]. The study highlights that Shubat exhibits a higher and more varied bacterial diversity compared to Ayran (another type of fermented milk). This elevated diversity is primarily attributed to the use of raw camel milk and a prolonged fermentation process spanning several days, accompanied by regular mixing [56]. This traditional production method facilitates the continuous introduction of environmental microbes, resulting in a complex community structure that varies significantly by geographical region [56].

## 4. Role of LAB in Spontaneously Fermented Camel Milk: Functions, Challenges, and Applications

LAB are well known for their ability to produce substantial amounts of bioactive compounds during fermentation [31]. LAB are the dominant population in raw and fermented milk; they produce various antimicrobials such as organic acids and hydrogen peroxide, antifungal peptides and bacteriocins, and play a crucial role in food fermentation processes [57]. Additionally, LAB contributes to the flavor, texture, and nutritional value of fermented foods through the production of aroma components, the production or degradation of exopolysaccharides, lipids, and proteins, and the production of nutritional components such as vitamins. In addition, they contribute to the inhibition of spoilage and pathogenic microorganisms [58].

Yeasts present in fermented camel milk are responsible for alcoholic fermentation, producing carbon dioxide and ethanol. This metabolic activity contributes to the characteristic foaming and imparts a distinct pungent and refreshing flavor to the final product [35]. However, camel milk fermentation typically does not result in the formation of a firm curd, leading to a relatively weak texture. This is mainly attributed to the large size of casein micelles, the small size of fat globules, and the absence of β-lactoglobulin (β-lg) [59]. Furthermore, the physicochemical properties of camel milk, together with its microbiota, determine the functional characteristics of these traditional fermented products [37]. Traditional practices such as shaking are often applied to prevent fat aggregation and to promote continuous lactic acid production, thereby limiting the growth of spoilage microorganisms such as yeasts and molds [29]. During fermentation, a dynamic microbial shift is observed, characterized by a decrease in *Lactococcus* populations and a concurrent increase in *Lactobacillus*, contributing to the development of organoleptic and functional properties, as reported in Shubat [12]. Despite being produced through spontaneous fermentation, which is often associated with lower hygienic control, some studies have reported the absence of critical pathogens such as *Salmonella* and *Shigella* in Shubat [35]. This suggests that indigenous LAB may contribute to inhibiting pathogenic bacteria.

Furthermore, LAB isolated from traditional products such as Chal have been successfully used to ferment both camel and bovine milk under controlled conditions. These isolates produced functional products with significantly enhanced antioxidant activity (measured by DPPH and ABTS) and improved sensory properties. This highlights the potential of indigenous LAB as starter cultures for the development of health-promoting fermented dairy products [8].

## 5. Microbial Identification Approaches: Integrating Culture-Dependent and Culture-Independent Methods

Culture-dependent methods rely on the isolation and cultivation of microorganisms under laboratory conditions [33]. Culture-based methods are estimated to identify less than 1% of the total microbial diversity [39], highlighting their limitations in fully characterizing complex microbial communities. These methods fail to capture the complexity and interactions within microbial communities, providing only a partial view of their structure and function [60]. To overcome these limitations, culture-independent sequencing approaches have emerged. Metagenomics is defined as the direct genetic analysis of genomes contained within an environmental sample [61]. For instance, 16S rRNA gene sequencing involves PCR amplification of conserved regions of the 16S rRNA gene, enabling taxonomic identification of microbial communities. [62]. However, a limitation of this method is that annotation is based on the putative association of the 16S rRNA gene with taxa defined as Operational Taxonomic Units (OTUs). In general, OTUs are analyzed at the phyla or genera level and can be less precise at the species level [63]. Consequently, whole-genome shotgun (WGS) sequencing represents an alternative approach that enables more accurate taxonomic resolution at the species level. However, this method is more costly and requires more complex data analysis [64]. These approaches have been widely applied in fermented dairy microbiome studies. High-throughput sequencing has uncovered an unprecedented variety of microbes, reinforcing the vital role of laboratory cultivation in downstream investigations. As a result, culture-dependent techniques have seen a resurgence in the study of bacterial communities [65].

The integration of culturomics and metagenomics represents a promising strategy due to their complementary strengths. While culture-independent approaches enable rapid and comprehensive detection of microorganisms, including those in a viable but non-culturable (VBNC) state, conventional culture methods remain essential for quality assurance and the isolation of viable strains. Therefore, a combined approach is necessary, while using genomic tools to explore microbial potential and culture-dependent techniques to validate viability and obtain live isolates for industrial applications [66].

In this context, culture-independent approaches have provided valuable insights into the microbiology of fermented camel milk. For example, the metagenomic mining of Shubat confirmed the low prevalence of antibiotic resistance genes (ARGs). This finding is crucial for identifying safe microbial strains, especially given the global rise in antibiotic resistance among many pathogens [30]. In addition, metagenomic analysis has identified biosynthetic gene clusters associated with secondary metabolites, including bacteriocins, as well as other compounds such as terpenes and furans. These findings highlight traditional fermented milk as a valuable reservoir of novel lactic acid bacteria and bioactive compounds with antimicrobial and flavor-enhancing potential [30]. Furthermore, the detection of non-conventional genera such as *Acinetobacter*, *Citrobacter*, and *Moraxella osloensis* is scientifically significant. These organisms are typically classified as environmental microbes, and their presence reflects the open and dynamic nature of traditional fermentation systems. Moreover, their detection underscores the sensitivity of high-throughput sequencing (HTS) in identifying microorganisms that may not be recoverable using standard culture techniques [56]. Table 2 summarizes some of the culture-dependent and -independent methods used to study the microbial community in fermented camel milk, highlighting some of their benefits and limitations.

## 6. Probiotic Lactic Acid Bacteria

Lactic acid bacteria isolated from raw and traditional fermented camel milk from different geographical regions can demonstrate substantial probiotic potential based on laboratory-based assays. These studies identified multiple genera, including *Lactobacillus*, *Enterococcus*, *Lactococcus*, *Pediococcus*, and *Leuconostoc*, with strains showing probiotic potential [70,71,72]. Most of these strains were able to survive low pH values (simulating gastrointestinal environments), exhibited substantial antimicrobial activities, including against pathogens, promoted strong immune responses, hydrolyzed bile and showed significant antioxidant activities while lacking hemolytic activities and virulence factors (Table 3). However, there are few in vivo studies on the use of these groups as probiotics, highlighting the need for more research to validate their probiotic potential in living systems. A summary of the different probiotic activities observed in some of the microbial isolates from raw and fermented milk is presented in Table 3.

## 7. Potential Standardization and Commercialization of Fermented Camel Milk

While not as widely consumed as bovine milk, camel milk is commercially available. For example, in Australia, the Humpalicious group sells fresh pasteurized Humpalicious Camel Milk [81]. Desert Farms in the US also sells raw and pasteurized milk [82]. However, the Camwell group in Mongolia commercially produces and sells fresh and fermented camel milk internationally [83].

In general, the standardization and commercialization of traditionally fermented camel milk will involve several key processes. The first step involves quality control of the milk to ensure that it is sourced from healthy camels and collected mechanically using hygienic milking practices. The collected raw milk should be tested to ensure that it meets the required quality standards regarding microbial load, nutritional and physicochemical characteristics. These steps would reduce the risk of bovine milk adulteration and excessive microbial contamination, sometimes associated with hand-milking [84]. The milk should be stored and appropriately transported to a milk processing unit (cold-chain management), where secondary testing and processing would take place.

**Figure 1:**
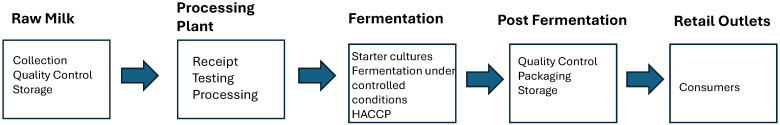
Schematic representation of the standardization and commercialization pathway for traditionally fermented camel milk.

To obtain a product with a consistent flavor and composition, the fermentation process of the milk must be standardized using developed starter cultures. The fermentation process should be carried out under controlled conditions, in contrast to the spontaneous traditional fermentation process, which often results in products of varying quality and flavor. It would be necessary to develop hazard and critical control points for camel milk production and fermentation to ensure product quality and safety. Finally, issues of shelf life, labeling, packaging, advertisement, transportation (under temperature control), distribution for retail sales and traceability will have to be addressed. A brief overview of this process is presented in Figure 1.

## 8. Conclusions

This review has shown that spontaneously fermented camel milk products, such as Gariss, Shubat, Chal, Dhanaan, and Suusac (or Suusa), due to their favorable nutritional profiles, are widely consumed by pastoral communities in different parts of the world. This fermentation is mediated by a complex group of microorganisms and influenced by factors such as the indigenous microbial composition of the milk, geographical locations, seasons and traditional fermentation practices. Both culture-dependent and -independent studies have shown that lactic acid bacteria are the main drivers of camel milk fermentation, playing important roles in the development of desirable sensory and organoleptic properties and in improving shelf life through acidification. In addition, yeasts were shown to contribute to milk fermentation, especially in products undergoing lactic–alcoholic fermentation. However, its microbial community composition is variable due to differences in fermentation and hygiene practices, and the use of different research methodologies limits cross-study comparability. The detection of opportunistic/contaminating pathogens in some of the traditional fermented milks highlights an important food safety issue that warrants further investigation. Additionally, future studies using multi-omics approaches are critical for improving microbial characterization and linking microbial presence to function. Further studies on the development of the probiotic potential of these microbial groups are needed. Overall, while fermented camel milk holds considerable potential for wider acceptance and commercialization, addressing safety challenges and improving microbial standardization is essential for this to occur.

## Figures and Tables

**Table 1:** The traditional fermented camel milk products, their country of origin, identification methods, and identified microorganisms.

Product	Country	LAB (Dominant Genera)	Yeasts and Molds	Potential Pathogens	Key Observation	Method	pH	Titratable Acidity (%)	Fermentation Time and Temperature	Ethanol Content (%)	Refs.
Lfrik	Morocco	*Lactococcus*, *Lactobacillus*, *Streptococcus*, *Leuconostoc*	Yeasts (6–7.5 log) and molds (~1 log)	Not detected	LAB-dominated fermentation	CD	4.7–5.9	0.32–0.50	Ambient temperature for up to 12 h	NR	[25]
Gariss	Sudan	*Lactobacillus*, *Streptococcus*, *Lactococcus*, *Leuconostoc*	Yeasts (~6–7.7 log)	*Streptococcus* infantarius (gtf+)	LAB diversity with occasional risk	CD + CI	3.4–5.1	1.68–1.85	Ambient temperature for up to 72–96 h	1.32–1.46	[7,26,27,28,29]
Suusac (or Suusa)	Somalia	*Lactobacillus*, *Lactococcus*, *Leuconostoc*	*Candida*, *Geotrichum*, *Rhodotorula*	*Klebsiella*, *E. coli*, *Shigella*, *S. aureus*; Coliforms	Hygiene-dependent variability	CD	4.3–5.0	Up to 1.2	Ambient temperature for up to 48–96 h	NR	[30,31,32,33]
Dhanaan	Ethiopia	*Lactobacillus*, *Streptococcus*, *Lactococcus*	Yeasts and molds (~7 log)	*Klebsiella*, *E. coli*, *Salmonella*, *Shigella*, *Cronobacter*	Mixed microbiota with safety concerns	CD + CI	4.0–4.2	1.5–1.75	Ambient temperature for up to 72 h	NR	[2,9,34]
Chal	Iran	*Lactobacillus*, *Leuconostoc*, *Weissella*	Diverse yeasts	Not reported	Yeast-rich fermentation	CD	4–6	Up to 0.4	Ambient temperature for up to 48 h	0.4–0.7	[8,35]
Shubat	Kazakhstan	*Lactobacillus*, *Lactococcus*, *Leuconostoc*	*Kluyveromyces*, *Candida*	Not reported	Confirmed by metagenomics	CI + MG	3.7–4.1	0.17–0.24	Ambient temperature for up to 48 h	0.6–2.8	[12,36,37,38,39]

Note: CD = culture-dependent, CI = culture-independent and MG = metagenomic.

**Table 2:** The advantages and disadvantages of culture-dependent and culture-independent approaches.

Approach	Technique	Advantages	Disadvantages	Refs.
Culture-Dependent	Plate Count: Target microbial groups (such as lactic acid bacteria, molds and pathogens in milk) are serially diluted and plated on general and multipurpose media such as Plate Count Agar (PCA), Nutrient Agar (NA), MRS Agar (de Man, Rogosa and Sharpe), VRBA (Violet Red Bile Agar) and Sabouraud Dextrose Agar (SDA). Isolation, Purification and Identification: Target microbial groups can be purified before being identified using microscopic and biochemical tests. MALDI-TOF-MS: Use of bacterial protein spectral fingerprinting for identification purposes	Typically low-cost, easy to carry out and with limited substantial technical expertise needed. Enumeration of viable microbial groups in raw and fermented milk. Identification of isolates, including pathogens in fermented milk. Culturable isolates can be obtained and used for downstream activities (species/strain typing and characterization, starter cultures for fermentation, probiotic use for milk fortification, etc.). Testing for specific activities such as antimicrobial activities, antibiotic resistance and production of bioactive compounds. MALDI-TOF allows for rapid and accurate identification of isolates	Only culturable microbial groups can be detected. Time-consuming. May favor fast-growing microbial groups. Slow-growing bacterial groups can be missed. Limited/poor resolution of closely related taxa and rare groups. Accuracy of identification can be low, especially if, as with MALDI TOF MS, the database is poorly curated.	[4,67,68,69]
Culture-Independent	Polymerase Chain Reactions (PCR): Endpoint, real-time quantitative PCR (qPCR). Target DNA can be amplified and quantified. [50] 16S rRNA Amplicon and ITS Sequencing: For profiling and identification of the microbial (LAB, molds, etc.) communities in raw and fermented milk. Next-Generation Sequencing: 16S rRNA gene-based, shotgun and whole-genome metagenomics and metatranscriptomics. Applied for qualitative and quantitative analysis of the microbial community in raw and fermented milk.	Rapid, reliable, sensitive and accurate identification of isolates (target groups and pathogens). Comprehensive/detailed overview of microbial community (culturable and non-culturable groups) diversity. Functional characterization of target microbial groups. Excellent for high-resolution community profiling, microbial shifts and succession.	Comparatively higher cost for reagents, equipment and bioinformatic analysis. Substantial scientific and technical expertise required. PCR amplification and database bias can occur. No information on the viability of target microbial groups (DNA could be from dead or living cells).	[11,12,17,50,56]

**Table 3:** Probiotic properties of lactic acid bacteria isolated from raw and fermented camel milk products.

Probiotic Property	Probiotic Activities	Microbial Groups	References
Antimicrobial activities	Inhibition of bacteria (e.g., *Staphylococcus aureus*, *Escherichia coli*, *Listeria monocytogenes*, *Helicobacter pylori* and *Salmonella typhi*) and fungal pathogens (e.g., *Trichophyton mentagrophytes* and *Candida albicans*) and spoilage groups. Improves the safety and nutritional profile and shelf life of milk.	*Lactobacillus* sp. *Lactobacillus helveticus*, *Limosilactobacillus reuteri*	[70,73,74,75]
Promotion of strong immune responses	Increased synthesis of polyclonal antibodies such as IgG, IgM and IgA. Improved mucosal responses with the expression of TLR2 (Toll-like receptor 2) and IFNγ (Interferon-gamma) mRNA (in test animals)	*Lactobacillus* sp.	[73]
Bile salt tolerance	Tolerance allows the microbial group to survive and carry out any probiotic function in the GIT (small intestine)	*Lactobacillus* sp., *Enterococcus lactis*, *L. plantarum*	[73,74]
Acid tolerance	Surviving the acidity of the stomach (pH 2) is critical to any potential beneficial activity promoting gut health.	*Lactobacillus* sp. and *L. helveticus*	[70,71,76]
Cholesterol reduction	Reduces the risk of stroke and cardiovascular issues	*L. lactis*, *L. plantarum*, *L. casei*, *Streptococcus* sp. and *Enterococcus* sp.	[71,76,77]
Non-hemolytic and/or susceptible to antibiotics	Improves the milk’s safety and reduces the risk of antibiotic resistance development	*L*. *plantarum*, *L. lactis*, *Leuconostoc mesenteroides*, *L. helveticus* and *Pediococcus* sp.	[23,70,74]
Auto-aggregation and hydrophobicity.	Promotes adhesion to the intestinal wall, necessary for probiotic activity and pathogen exclusion	*Lactobacillus* sp.* L. plantarum*, *L. casei*, *L. gasseri* and *Pediococcus pentosaceus*	[23,74,78,79]
Antioxidant activities	Promotes gut health and cellular function through DPPH free radical scavenging	*Lactobacillus* sp.* L. plantarum*, *Enterococcus faecium* and *L. lactis*	[79,80]

## Data Availability

No new data were created or analyzed in this study. Data sharing is not applicable to this article.
